# Primary Cytomegalovirus Infection in Seronegative Kidney Transplant Patients Is Associated with Protracted Cold Ischemic Time of Seropositive Donor Organs

**DOI:** 10.1371/journal.pone.0171035

**Published:** 2017-01-27

**Authors:** Fabian Schlott, Dominik Steubl, Dieter Hoffmann, Edouard Matevossian, Jens Lutz, Uwe Heemann, Volker Hösel, Dirk H. Busch, Lutz Renders, Michael Neuenhahn

**Affiliations:** 1 Institute for Medical Microbiology, Immunology and Hygiene, Technische Universität München, Munich, Germany; 2 German Center for Infection Research (DZIF), Partner Site Munich, Munich, Germany; 3 Department of Nephrology, Klinikum Rechts der Isar, Technische Universität München, Munich, Germany; 4 Institute of Virology, Technische Universität München/ Helmholtz Zentrum München, Munich, Germany; 5 Department of Surgery, Transplantationszentrum München Klinikum Rechts der Isar, Technical University München, Munich, Germany; 6 Section of Nephrology, I. Department of Medicine, Johannes-Gutenberg University Mainz, Mainz, Germany; 7 Technical University Munich, Chair of Biomathematics, Garching, Germany; University of San Francisco, UNITED STATES

## Abstract

Human Cytomegalovirus (CMV) can lead to primary infection or reactivation in CMV-seronegative or -seropositive kidney transplant recipients, respectively. Complications comprise severe end-organ diseases and acute or chronic transplant rejection. Risk for CMV manifestation is stratified according to the CMV-IgG-serostatus, with donor+/recipient- (D+/R-) patients carrying the highest risk for CMV-replication. However, risk factors predisposing for primary infection in CMV-seronegative recipients are still not fully elucidated. Therefore, we monitored D+/R- high-risk patients undergoing kidney transplantation in combination with antiviral prophylaxis for the incidence of CMV-viremia for a median follow-up time of 784 days (156–1155 days). In this period, we analyzed the functional CMV-specific T cell response by intracellular cytokine staining and CMV-serology by ELISA. Only four of eight D+/R- patients developed clinically relevant CMV-viremia followed by seroconversion. Viremia triggered expansion of functional CMV-specific T cells correlating with protection against secondary CMV-reactivations. In contrast, all other patients remained permanently aviremic and showed no immunological correlate of infection after discontinuation of antiviral prophylaxis for up to three years. Comparing cold ischemic times (CIT) of viremic (median = 1020 min; 720–1080 min) and aviremic patients (median = 335 min; 120–660 min) revealed significantly (p = 0.0286) protracted CIT in patients with primary CMV-infection. Taken together, primary CMV-infection affects only a subgroup of D+/R- patients correlating with length of CIT. Therefore, patients with extended CIT should be thoroughly monitored for CMV-replication well beyond discontinuation of antiviral prophylaxis. In contrast, patients with short CIT remained permanently uninfected and might benefit from shorter prophylactic treatment.

## Introduction

Human Cytomegalovirus (CMV) is one of the major infectious complications after kidney transplantation. Primary infection in seronegative patients receiving seropositive grafts or reactivation in seropositive recipients can lead to CMV disease with serious end-organ manifestations (e.g. pneumonitis/ colitis) or graft failure [1–4]. Therefore, control of de-novo infection in seronegative or reactivation in seropositive patients is of major importance. Based on the CMV-IgG-serostatus of donor (D) and recipient (R) a high (D+/R-), intermediate (D+/R+ and D-/R+) and low (D-/R-) risk constellation has been defined [5–7]. For high risk constellations, prophylactic treatment with antiviral agents such as Valganciclovir for three, and sometimes for six months is recommended [5, 7, 8]. Although the rate of clinical CMV manifestations can be reduced by prophylaxis, the use of antiviral drugs is associated with unfavorable allograft long-term-outcome [9, 10]. In consequence, avoidance of unnecessary prophylactic treatment in D+/R- patients would be desirable.

The expansion of CMV-specific CD8^+^ T cells plays a central role for the control of CMV in solid organ transplantation patients [7, 11, 12]. Therefore, we monitored D+/R- kidney transplantation patients with regard to the development of CMV-specific T cell immunity in order to establish a risk-based approach for the appropriate duration of antiviral prophylaxis in T cell immune vs non-immune patients. Indeed, the majority of D+/R- patients with detectable CMV-viremia following a 3-month CMV prophylaxis established a CMV-specific CD8^+^ T cell response and were protected further on from viral replication. Interestingly, half of the examined D+/R- patients lacked CMV-specific immunity but did not suffer from viral replication indicating that primary CMV-infection might depend on still unknown risk factors.

We identified here cold ischemic time (CIT) as a potential risk factor for primary CMV-infection in D+/R- renal transplant patients.

## Materials and Methods

### Patient characteristics

Between 2011 and 2013, we included 8 D+/R- high risk patients who received a kidney (n = 7) or kidney/ pancreas (n = 1) transplant as a subgroup of a larger observational trial at the University Hospital Klinikum rechts der Isar, Munich, Germany. The study was approved by the ethics committee of the Faculty of Medicine, Technical University Munich and in accordance with the declarations of Helsinki and Istanbul. All enrolled patients had given their written informed consent. None of the transplant donors were from a vulnerable population and all donors or next of kin provided written informed consent that was freely given. The study was based on an observational cohort study concept. All patients were treated prophylactically with 450 mg Valganciclovir three times per week for three months after transplantation. Valganciclovir dosage was adapted according to the estimated Glomerular filtration rate (eGFR) as recommended by the product information. Immunosuppressive therapy consisted of tacrolimus, mycophenolate and steroids. 6 patients received induction therapy intraoperatively with rabbit anti-thymocyte globulin (ATG). Screening for CMV-infection was performed every two weeks in the first three months after transplantation and thereafter every four weeks using CMV polymerase chain reaction (PCR). We amplified a conserved part of the CMV polymerase gene using a validated in house protocol. Starting with 50°Celcius for 2 minutes and 95°Celcius for 10 minutes, reactions were cycled 45 times at 95°Celcius for 15 seconds and 60°Celcius for 1 minute. We participate in nationwide round robin tests every 6 months and thus ensure correct CMV DNA quantitation. Median follow-up was 784 days (156–1155 days). In order to detect CMV-specific immune responses, approximately 30 ml of NH_4_-heparinized blood was collected before transplantation and on day 28, 90, 180, 270 and 360 days after transplantation.

### Plasma collection and peripheral blood mononuclear cells (PBMC) isolation/ cryopreservation

Plasma was isolated form heparinized whole blood by centrifugation at 700g for 10 minutes and was then cryopreserved at -80°Celsius. Afterwards, PBMCs were isolated from blood diluted 1:1 with phosphate-buffered saline (PBS, Biochrom AG, Berlin, Germany) using Ficoll (Biocoll, Biochrom AG) differential centrifugation at 700g for 25 minutes. After separating the different blood components, PBMCs were transferred to a new falcon and washed twice with PBS, respectively RPMI (Sigma-Aldrich, Taufkirchen, Germany). For long-term storage PBMCs were cryopreserved in liquid nitrogen using a 90% fetal calf serum (FCS, Biochrom AG) and 10% dimethyl sulfoxide (DMSO, Sigma-Aldrich) solution.

### Detection of CMV-specific antibodies

In order to detect CMV-specific antibody responses before and after transplantation the previously collected plasma was used. For the detection of CMV-IgG and—IgM antibodies the CMV-IgG-ELA Test PCS and CMV-IgM-ELA Test PCS (both Medac, Wedel, Germany) were used in accordance with the manufacturer’s instructions. For the detection of CMV-IgG and—IgM of aviremic patients the Architect c4000 (Abbott GmbH & Co. KG, Wiesbaden, Germany) and CMV-IgG/ CMV-IgM reagents (Abbott GmbH & Co. KG) were used.

### QuantiFERON CMV

The assay was performed according to manufactures instructions. In brief: QuantiFERON CMV tubes (Qiagen, Hilden, Germany) were filled with 1ml of whole blood. Tubes were shaken and incubated for 18–24 hours at 37°Celsius. Supernatant was harvested and interferon-γ (IFNγ)-release (IU/ml) was measured using the QuantiFERON CMV ELISA (Qiagen, Germany) according to manufacturer’s instructions.

### Intracellular cytokine staining (ICS) for the detection of functional CMV-specific T cells

Cryopreserved PBMCs were thawed, rested for 18 hours in RPMI/ 10% FCS (2x10^6^ cells/ml) and stimulated with 2μg/ml of pools of peptides (15mer with 11aa overlap) covering the whole immediate-early protein 1 (IE-1) or the whole 65 kDa phosphoprotein (pp65; JPT Peptide Technologies GmbH, Germany) in the presence of 1 μg/ml anti-CD28 (BD Biosciences, San Jose, USA) and 1 μg/ml anti-CD49d (BD Biosciences) costimulatory antibodies for 1 hour at 37°/ 5% CO_2_. Then 0.01 μg/μl Brefeldin A (Sigma-Aldrich) was added and incubated for 3.5 hours. For live dead discrimination, cells were stained for 10 minutes on ice with 2 μg/ml ethidium bromide monoazide (EMA, Sigma-Aldrich). Surface staining was performed for 30 minutes on ice with anti-CD8 PerCP (BD Biosciences), anti-CD3 eFluor 450 (eBioscience, San Diego, USA). Afterwards, cells were permeablized/fixed for 20 minutes on ice using BD^™^ Cytofix/Cytoperm kit (BD Biosciences), and for ICS, cells were incubated for 30 minutes on ice with IFNγ Alexa Fluor^®^ 700 (eBioscience). Cells were acquired using a BD^™^ LSR II (BD Biosciences) and analyzed by FlowJo (FlowJo, LLC, Ashland, USA) software.

### Quantification of absolute cell counts and statistical analysis

For the calculation of absolute CMV-specific T cells the BD^™^ Trucount kit (BD Biosciences) was used. Statistical analysis were performed with the Mann-Whitney *U* test and calculated by GraphPad Prism 5 (GraphPad Software, La Jolla, USA) for Windows.

## Results

Eight CMV-seronegative patients received a graft from a CMV-seropositive donor during the study period and were therefore at high risk for primary CMV-infection. After transplantation these D+/R- patients were intensively monitored for a median time of 784 days (156–1155 days). The demographic parameters of these patients are summarized in S1 Table.

### Viremic D+/R- patients show severe CMV complications but generate a CMV-specific immune response

**Fig 1:**
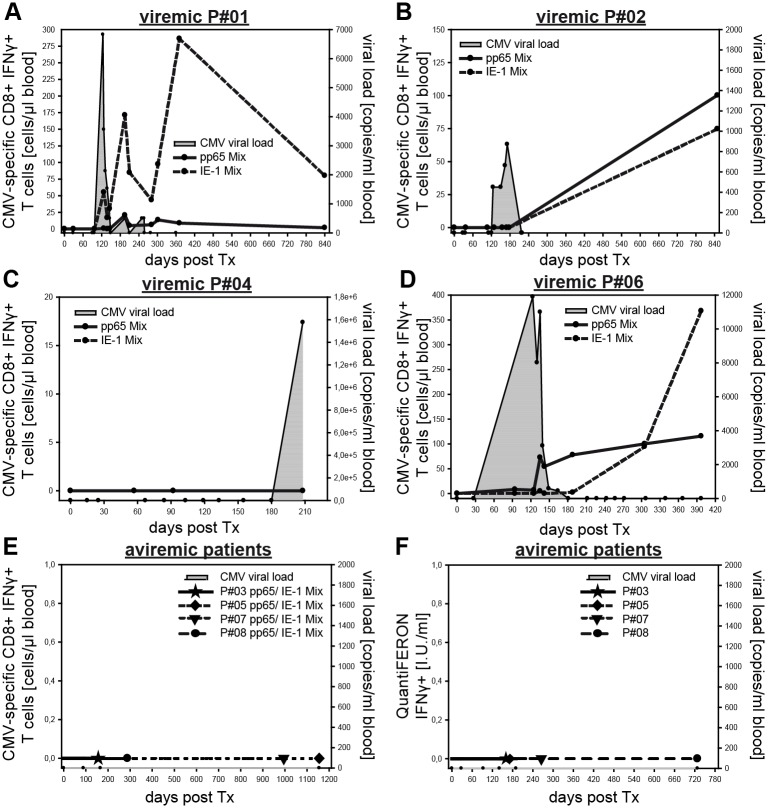
Longitudinal monitoring of CMV-specific immunity reveals heterogeneous risk for primary CMV-infection in D+/R- renal transplant recipients. High risk D+/R- recipients were observed for a median follow-up time of 784 days (156–1155 days). CMV-viremia (grey area) was measured by quantitative PCR, CMV-specific T cells were quantified by IFNγ secretion after restimulation with CMV-specific peptide mixes in ICS. Viremic patients are separately shown in (A)—(D), kinetics of CMV-pp65- (solid line) or CMV-IE-1- (dashed line) specific CD8^+^ T cells are indicated. Patients without viremia are shown together in (E), last time points of negative T cell screenings are indicated. (F) Quantification of IFNγ secretion using the CMV-QuantiFERON assay in patients without viremia.

4 of 8 patients developed manifest CMV-infections after finishing prophylactic treatment with Valganciclovir. 3 out of these 4 patients developed CMV-specific immunity during infection, which protected against further CMV-replications (Fig 1). Patient #01, with a negative CMV PCR result on postoperative day (POD) 96, developed acute CMV-infection on POD 124 and suffered from CMV-associated pneumonitis, which was treated by intravenous ganciclovir in an ICU setting. On POD 127 we detected CMV-specific T cells directed against pp65 (0.67 cells/μl blood) and IE-1 (55.2 cells/μl blood) virus epitopes accompanied by seroconversion for CMV-IgM/IgG (Table 1). After strong proliferation of CMV-specific CD8^+^ T cells (pp65 Mix: POD 195, 20.6 cells/μl blood; IE-1 Mix; POD 371, 286.3 cells/μl blood) and control of viremia patient #01 remained protected from further CMV-reactivations (Fig 1A). Similarly, patient #02 showed CMV-replication between POD 125 and POD 171 (max. 872 copies/ml blood). On POD 177 CMV-IgG was measurable (Table 1) and viremia was controlled on POD 224. Proliferation of functional CMV-specific T cells directed against pp65 (100.2 cells/μl blood) and IE-1 (74.7 cells/μl blood) epitopes could be confirmed on POD 849 (Fig 1B). Patient #04 showed a delayed CMV syndrome (fever and leuko-/ thrombocytopenia) together with CMV-IgM seroconversion (Table 1) and high grade CMV-viremia (>1.5x10^6^ copies/ml blood) on POD 208. The patient died already five days later due to a *S*. *aureus*-associated sepsis without a detectable CMV-specific T cell response (Fig 1C). Patient #06 showed a severe CMV-infection on POD 123 with 11900 copies/ml. He suffered from CMV-associated hepatitis, for which intravenous ganciclovir was administered. We detected CMV-specific antibodies on POD 94 (CMV-IgM) and a class switch to CMV-IgG on POD 135 (Table 1). CMV-specific T cells were detected on POD 94 directed against pp65 (8.3 cells/μl blood) and IE-1 (0.04 cells/μl blood) epitopes. Expansion of functional CMV-specific T cells correlated with control of CMV viremia in D+/R- patients (Fig 1D).

**Table 1:** CMV-IgM and -IgG serostatus of viremic and aviremic patients.

Patient No.	Days post Tx	CMV IgM serostatus	Days post Tx	CMV IgG serostatus
**Viremic patients**				
1	127	positive	127	positive
2	177	*negative*	177	positive
4	208	positive	208	*negative*
6	94	positive	135	positive
**Aviremic patients**				
3	165	*negative*	165	*negative*
5	1155	*negative*	1155	*negative*
7	995	*negative*	995	*negative*
8	728	*negative*	728	*negative*

### Aviremic patients lack CMV-specific immunity

Since the other four D+/R- patients remained aviremic after termination of Valganciclovir, we expected them being protected by CMV-specific T cells that could have been induced by subclinical CMV replication during antiviral prophylaxis. However, we could surprisingly not find any signs of CMV-specific T cell immunity during the whole follow-up period in these patients, neither measured by ICS (Fig 1E) nor by the CMV-QuantiFERON assay (Fig 1F). Furthermore, to exclude that transient low level CMV viremia was not detected by PCR, we additionally screened the patients for CMV-IgM/IgG antibodies. Including very late time points (median = 861.5 days), all aviremic patients remained CMV-seronegative over the whole time course (Table 1). Taken together, despite their D+/R- high risk constellation, we found in these patients no correlate of CMV-infection.

### Risk factors predisposing for primary CMV-infection

**Fig 2:**
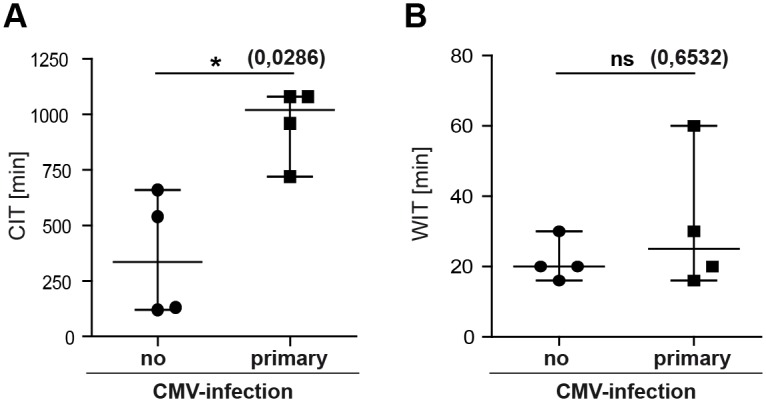
Cold ischemic time is correlated with primary CMV-infection. (A) Comparison of cold ischemic times in viremic (median = 1020 min; 720–1080 min) and aviremic patients (median = 335 min; 120–660 min). (B) Comparison of warm ischemic time (WIT) in viremic (median = 25 min; 16–60 min) and aviremic patients (median = 20 min; 16–30 min). Statistical analysis were performed with the Mann-Whitney *U* test. * = p < 0.05.

It is known, that inflammatory cytokines like tumor necrosis factor (TNF) can trigger CMV recurrence from latency [13, 14], and increased levels of inflammatory cytokines can be measured in reperfused donor kidneys (ischemic reperfusion injury) [15, 16]. Notably, aggravated reperfusion injury is associated with prolonged storage of the organ in cold solution, termed cold ischemic time (CIT) [15]. Furthermore, extended warm ischemia time (WIT) is known to cause serious graft damage, which might also induce CMV-replication [13, 17]. Therefore, we tested if CIT or WIT might influence CMV recurrence from latency leading to primary CMV-infection of D+/R- high risk patients. All patients with viremia (median = 1020 min; 720–1080 min) and seroconversion had a significantly longer CIT than patients without infection (median = 335 min; 120–660 min; p = 0.0286; Fig 2A), while warm ischemic time (WIT) was comparable between both groups (Fig 2B). Furthermore, we detected a monotone statistical relationship (Spearman r = 0.9315) between prolonged CIT and peaks of viral titers (S1A Fig). In line with this, there also seems to be a positive relationship between length of CIT and severity of CMV-induced disease (S1B Fig). In order to exclude other factors possibly influencing the risk for an acute CMV-infection, we compared age, induction therapy, immunosuppressive medication, and human leukocyte antigen match in both groups [7, 18]. None of these parameters differed significantly between infected and uninfected D+/R- patients. T cell depletion after ATG treatment has been described as a risk factor for CMV manifestations in kidney transplant recipients [19]. Since primary CMV-infection occurred only in ATG-treated patients, we analyzed additionally the absolute number of CD3^+^ and CD8^+^ T cells, respectively, but could not detect any significant differences between viremic and aviremic patients (S2 Fig), indicating that the immunological scar left by the applied ATG doses was more subtle and absolute CD3^+^/CD8^+^ T cell numbers cannot be used as suitable biomarker. In conclusion, prolonged CIT is a factor which is associated with primary CMV-infection in D+/R- recipients.

## Discussion

Cytomegalovirus infection is a common complication in solid organ transplantation, especially in D+/R- high risk patients, and correlates negatively with patient and graft survival [1, 2]. Therefore we analyzed potentially predisposing factors for primary CMV-infection in D+/R- patients and identified CIT as a relevant risk factor. This is in line with previous findings characterizing CIT to play an important role in CMV-infection of D+/R+ and D-/R+ liver transplant recipients [20].

Interestingly, four patients in our cohort with CIT < 12 hours did not show any correlate of CMV-infection implicating that under the described circumstances the risk of primary infection from latently CMV-infected organ donors can be significantly reduced. Importantly, we found no evidence for a contribution of preformed CMV-specific memory T cells as recently described in seronegative recipients [21]. In contrary, neither CMV-specific T cells nor CMV-IgM/IgG could be detected over the whole time course of follow-up. We cannot exclude that CMV persisted in a non-replicative dormant state within the donor organ. However, local CMV replication in the donor organ as well as asymptomatic infection of other donor tissues should have led to CMV-specific traces in cellular or humoral host immunity. Therefore, a successful CMV infection of these aviremic D+/R- recipients is very unlikely. Even though the exact molecular mechanism of CMV prevention remains at this point still elusive, we hypothesize that CMV persists in donor kidneys with short CITs, presumably little reperfusion injury and consecutively low levels of inflammatory cytokines primarily in a non-replicative, latent state [16]. Latently infected cells like monocytes [13], which have a short half life time, would then be ultimately replaced by uninfected recipient cells preventing CMV transmission. Besides monocytes, also dendritic cells (DC) are potential CMV genome carriers even though latency in these terminally differentiated cells has not yet been demonstrated. However, it was recently shown in mice that donor-derived DCs are also rapidly replaced by recipient DCs after kidney transplantation [22]. Still, the precise mechanistic role of different cellular subsets in viral transmission requires careful analysis of kidney biopsies and needs to be addressed in future studies. Clinically, it remains to be determined whether in this newly identified patient subgroup a 3 month Valganciclovir prophylaxis is required to inhibit viral transmission or if it might be justified to discuss a shorter Valganciclovir prophylaxis, still sufficient to prevent CMV-infections in high risk kidney transplantation recipients with short CIT.

In contrast, if CMV propagated within D+/R- patients with long CITs, it mostly caused clinically relevant complications. Whether ATG treatment, which all infected patients had received, influences in combination with protracted CIT the risk of infection by either depletion of CMV-specific, donor-derived kidney-resident memory T cells [23, 24] or unspecific inflammatory effects remains to be determined. Overall, patients with protracted CIT could benefit of a 200 days Valganciclovir prophylaxis, as it was shown that the incidence of CMV-associated disease in D+/R- kidney recipients was reduced by prolonged prophylactic treatment [8]. Alternatively, a prolonged phase of intensive preemptive screening could be preferable to reduce fatal CMV complications. This approach would allow simultaneously a controlled triggering of CMV-specific T cells leaving protective immunity, which could be ultimately confirmed by one of the available T cell assays.

In summary, these unexpected results can influence the discussion on preemptive/ prophylactic treatment of CMV after kidney transplantation in D+/R- patients and imply, if confirmed in larger cohorts, the requirement of a risk stratification in the, thus far, homogenous CMV “high risk” group.

## Supporting Information

S1 Fig CIT correlates with maximum CMV viral load and is associated with disease severity. (A) Correlation of CIT and maximum detectable viral load (Pearson r = 0.4459; Spearman r = 0.9315). (B) Depiction of the positive relationship between CIT and severity of CMV complications.
(TIF)

S2 Fig Analysis of ATG effects on absolute CD3^+^ and CD8^+^ T cell numbers. Depicted are the individual absolute CD3^+^ (grey line) and CD8^+^ (black line) T cell numbers with regard to viral load (grey area) and ATG administration of viremic (A) and aviremic (B) high risk D+/R- recipients. Comparison of absolute CD3^+^ (C) and CD8^+^ (D) T cell numbers in blood samples of viremic (circle) and aviremic (square) recipients is shown at pre Tx, POD 90 and POD 180. Statistical analysis was performed with the Mann-Whitney *U* test.
(TIF)

S1 Table Demographic data of D+/R- study participants. (DOCX)
