# Supplementary figures and images for: Primary Cytomegalovirus Infection in Seronegative Kidney Transplant Patients Is Associated with Protracted Cold Ischemic Time of Seropositive Donor Organs
Source: PLoS One. 2017 Jan 27;12(1):e0171035. doi: 10.1371/journal.pone.0171035 (PMC5271354; doi:10.1371/journal.pone.0171035)

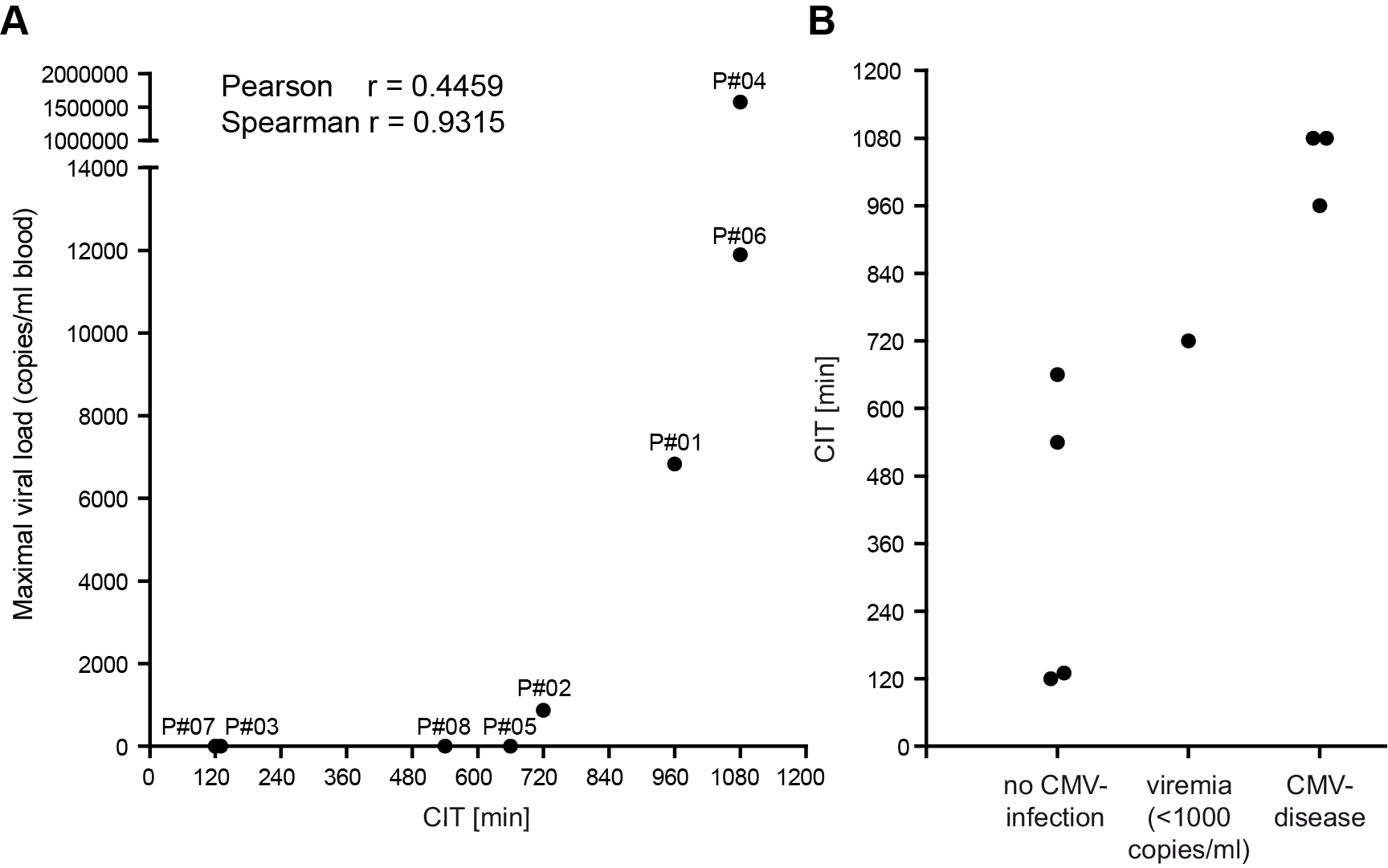

Supplement: S1 Fig — (A) Correlation of CIT and maximum detectable viral load (Pearson r = 0.4459; Spearman r = 0.9315). (B) Depiction of the positive relationship between CIT and severity of CMV complications. (TIF) [file pone.0171035.s001.tif]

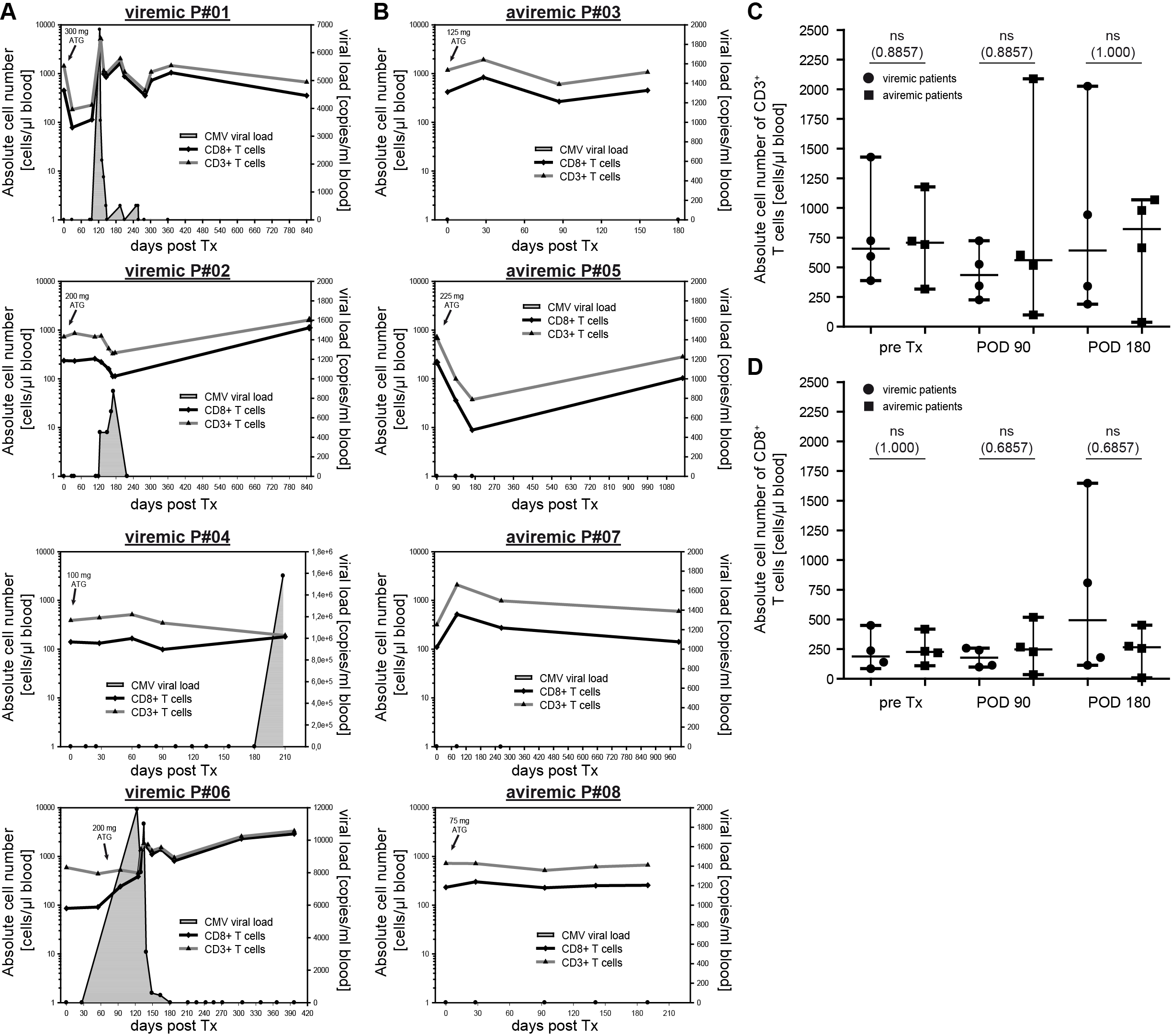

Supplement: S2 Fig — Depicted are the individual absolute CD3+ (grey line) and CD8+ (black line) T cell numbers with regard to viral load (grey area) and ATG administration of viremic (A) and aviremic (B) high risk D+/R- recipients. Comparison of absolute CD3+ (C) and CD8+ (D) T cell numbers in blood samples of viremic (circle) and aviremic (square) recipients is shown at pre Tx, POD 90 and POD 180. Statistical analysis was performed with the Mann-Whitney U test. (TIF) [file pone.0171035.s002.tif]
