# Supplementary material for: Primary Cytomegalovirus Infection in Seronegative Kidney Transplant Patients Is Associated with Protracted Cold Ischemic Time of Seropositive Donor Organs
Source: PLoS One. 2017 Jan 27;12(1):e0171035. doi: 10.1371/journal.pone.0171035 (PMC5271354; doi:10.1371/journal.pone.0171035)
Supplement: S1 Table — (DOCX) [file pone.0171035.s003.docx]

**Supplementary Table 1:** Demographic data of D+R- study participants

| No. | Gender | Age | Type of transplant | Number of transplantations | ATG induction (mg) | Cold-ischemia-time  (min) | Warm ischemia time  (min) | Underlying disease | Pre–transplant renal replace-ment therapy | CMV infection | CMV-associated disease |
| --- | --- | --- | --- | --- | --- | --- | --- | --- | --- | --- | --- |
| 1 | f | 56 | K/P | 1 | 300 | 960 | 60 | DN | HD | Yes | Pneumonitis |
| 2 | m | 54 | K | 2 | 200 | 720 | 20 | RN | HD | Yes | none |
| 3 | f | 49 | K | 1 | 125 | 130 | 20 | DN/HN | None | No | none |
| 4 | m | 40 | K | 1 | 100 | 1080 | 30 | DN | HD | Yes | CMV syndrome/ Sepsis |
| 5 | m | 53 | K | 1 | 225 | 660 | 30 | Fabry’s disease | HD | No | none |
| 6 | m | 62 | K | 1 | 200* | 1080 | 16 | ADPKD | HD | Yes | Hepatitis |
| 7 | f | 51 | K | 1 | - | 120 | 20 | MPGN | None | No | none |
| 8 | f | 52 | K | 2 | 75 | 540 | 16 | HUS | HD | No | none |

f = female; m = male; K/P = kidney/pancreas transplantation; K = kidney transplantation, ATG = rabbit anti thymocyte globulin; DN = diabetic nephropathy; RN=reflux nephropathy; HN = hypertensive nephropathy; ADPKD = autosomal-dominant polycystic kidney disease; MPGN = membrano-proliferative glomerulonephritis; HUS = hemolytic-uremic-syndrome; HD = hemodialysis; * = post-transplant ATG treatment preceding CMV replication
